# Peripheral Sensory Organs Contribute to Temperature Synchronization of the Circadian Clock in *Drosophila melanogaster*

**DOI:** 10.3389/fphys.2021.622545

**Published:** 2021-02-02

**Authors:** Rebekah George, Ralf Stanewsky

**Affiliations:** Institute of Neuro- and Behavioral Biology, Westfälische Wilhelms-Universität Münster, Münster, Germany

**Keywords:** circadian clock, temperature entrainment, chordotonal organ, antenna, rhodopsin, TRP channels, variant ionotropic glutamate receptors, period, timeless alternative splicing

## Abstract

Circadian clocks are cell-autonomous endogenous oscillators, generated and maintained by self-sustained 24-h rhythms of clock gene expression. In the fruit fly *Drosophila melanogaster*, these daily rhythms of gene expression regulate the activity of approximately 150 clock neurons in the fly brain, which are responsible for driving the daily rest/activity cycles of these insects. Despite their endogenous character, circadian clocks communicate with the environment in order to synchronize their self-sustained molecular oscillations and neuronal activity rhythms (internal time) with the daily changes of light and temperature dictated by the Earth’s rotation around its axis (external time). Light and temperature changes are reliable time cues (Zeitgeber) used by many organisms to synchronize their circadian clock to the external time. In *Drosophila*, both light and temperature fluctuations robustly synchronize the circadian clock in the absence of the other Zeitgeber. The complex mechanisms for synchronization to the daily light–dark cycles are understood with impressive detail. In contrast, our knowledge about how the daily temperature fluctuations synchronize the fly clock is rather limited. Whereas light synchronization relies on peripheral and clock-cell autonomous photoreceptors, temperature input to the clock appears to rely mainly on sensory cells located in the peripheral nervous system of the fly. Recent studies suggest that sensory structures located in body and head appendages are able to detect temperature fluctuations and to signal this information to the brain clock. This review will summarize these studies and their implications about the mechanisms underlying temperature synchronization.

## Introduction

Ever since Colin Pittendrigh described stable entrainment of *Drosophila pseudobscura* eclosion rhythms to temperature cycles, it was clear that this geophysical rhythm serves as a potent Zeitgeber, at least in poikilothermic animals (Zimmerman et al., 1968). Later studies showed that the adult activity rhythms of *Drosophila melanogaster* can also be synchronized by temperature cycles (Wheeler et al., 1993). Remarkably, the difference between the temperature “encoding” night and day can be as low 2–3°C, indicating that the fly clock is very sensitive to rhythmic temperature fluctuations (Wheeler et al., 1993; Chen et al., 2015). It was noted early on, that this apparent sensitivity to temperature changes presents some kind of paradox, considering the relative temperature independence of the free-running circadian period, known as temperature compensation (Zimmerman et al., 1968). Although theoretical considerations suggest that temperature entrainment and compensation properties are mechanistically linked (Pavlidis et al., 1968; Zimmerman et al., 1968), an alternative hypothesis is that they are based on largely independent processes. In support of this idea, all genetic variants that are known to affect temperature entrainment properties show normal temperature compensation (Glaser and Stanewsky, 2005; Wolfgang et al., 2013; Lee et al., 2014; Chen et al., 2015, see below).

The fly’s central pacemaker consists of 150 clock neurons; all of which express the key clock genes, and are named based on their anatomical distribution in the dorsal and lateral protocerebrum as well as for their relative sizes (reviewed in Hermann-Luibl and Helfrich-Förster (2015)]. Briefly, the lateral neurons consist of four subgroups known as the large and small ventrolateral neurons (l-LNv and s-LNv, respectively), the 5th small ventrolateral neuron (5th s-LNv), the dorsolateral neurons (LNd) and the lateral posterior neurons (LPN). An additional three subgroups of clock neurons are positioned dorsally in the fly brain and are known as the DN1 anterior and posterior (DN1a and DN1p, respectively), DN2 and DN3 clock neurons.

As it is known for entrainment to light:dark (LD) cycles (e.g., Kaneko et al., 2000), all of the 150 clock neurons in the fly brain synchronize to temperature cycles, both in constant darkness (DD) and in constant light (LL) (Yoshii et al., 2005, 2009; Gentile et al., 2013; Chen et al., 2015). For LD entrainment, light input into the clock neurons is mediated both by the visual system and by the blue light photoreceptor Cryptochrome (Cry), which is expressed in about 50% of the central clock neurons (Helfrich-Förster, 2020), showing that both peripheral sensory organs (i.e., the compound eyes) and endogenous clock neuronal light reception (Cry) contribute to entrainment. In principle, the same could apply for temperature entrainment, but it would formally also be possible that (a) temperature perception is exclusively mediated by thermosensors in the PNS, or (b) that the clock neurons are thermosensitive per se, either due to expression of a thermoreceptor, or because aspects of clock gene expression (e.g., mRNA splicing) are directly sensitive to temperature changes. In the following, we summarize what is currently known about temperature synchronization of the circadian clock in *D. melanogaster* with emphasis on the central brain clock, known to drive the activity rhythms of the fly.

## Direct Effects of Temperature on *per* and *tim* Splicing

Ambient temperature influences gene expression levels of hundreds of genes, including many genes with circadian functions (Boothroyd et al., 2007; Martin Anduaga et al., 2019). In addition, temperature modulates the amplitude and phase of *per* and *tim* expression, including the generation of alternatively spliced mRNAs [reviewed in Shakhmantsir and Sehgal (2019)]. Splicing of a *per* intron in the 3′-UTR (*dmpi8*) is increased at cooler temperatures and associated with higher daytime activity, while reduced *dmpi8* splicing at warm temperatures is correlated with delayed evening activity onset and extended day-time inactivity (siesta) (Majercak et al., 1999). Further studies implicated this *per* splicing event in the behavioral adaptation to hot and long summer days where less splicing leads to an increased siesta and a reduced risk of desiccation. Increased *per* splicing at cooler temperatures, most likely caused by less stringent hybridization of splicing factors with their target RNA, in turn support day activity as it is no longer harmful to the animal (Majercak et al., 1999, 2004; Collins et al., 2004). While these studies focused on potential effects of *per* splicing on *per* mRNA and PER accumulation to explain behavioral adaptation, a more recent study suggests a different molecular mechanism: cold-enhanced *dmpi8* splicing is not only correlated with enhanced *per* transcript levels, but also with increased transcript levels of the *daywake* gene (*dyw*). *dyw* encodes a juvenile hormone binding protein, which suppresses siesta behavior (Yang and Edery, 2019). The 3′ end of *dyw* overlaps with that of *per* and both genes are transcribed in opposite directions. It is therefore possible that the factors binding to the 3′ end of *per* and stabilize *per* mRNA, act *in trans* to stabilize *dyw* RNA. This intriguing mechanism would employ temperature and day-length dependent splicing of a core clock gene, but not its protein gene product (PER), to regulate a different gene (*dyw*) that directly influences siesta-promoting or -suppressing pathways (Yang and Edery, 2019).

The *tim* gene undergoes more complex temperature-dependent alternative splicing, which in contrast to *per* results in the generation of various truncated TIM proteins (Shakhmantsir and Sehgal, 2019). At warm temperatures (25–29°C) *tim* RNA undergoes an alternative splicing event, generating a transcript (named *tim-tiny* or *tim-M*), which retains an intron that introduces a premature stop codon (Shakhmantsir et al., 2018; Martin Anduaga et al., 2019). Rhythmic accumulation of *tim*^*tiny*^ during the warm phase of a 30°C:25°C TC in DD and LL correlates with reduced levels of full-length TIM. It appears that translation of *tim*^*tiny*^ mRNA is suppressed, resulting in extremely low levels of TIM^Tiny^ protein, while levels of TIM are reduced due to overall *tim* mRNA depletion caused by generation of *tim*^*tiny*^ transcripts (Shakhmantsir et al., 2018; Martin Anduaga et al., 2019). This mechanism presumably contributes to low TIM levels normally observed during the day, and to the delay between *tim* RNA and protein (Shakhmantsir et al., 2018). At cooler temperatures (18°C) *tim* RNA undergoes two different alternative splicing events resulting in the accumulation of *tim-cold*, *tim-short*, and cold (*tim-sc*) transcripts, which are translated into the respective truncated TIM proteins (Boothroyd et al., 2007; Martin Anduaga et al., 2019). Interestingly, flies in which generation of the *tim-sc* isoform is prevented (*tim*^*sc*^) restrict their behavioral activity to the light phase in LD 25°C, a behavioral pattern that is usually observed at 18°C in wild type flies (Majercak et al., 1999; Martin Anduaga et al., 2019). This shows that the *tim-sc* transcript is not required for behavioral adaptation to 18°C conditions, which presumably is mediated by increased levels of *tim-cold*. In support of this idea, *tim-cold* levels are strongly increased in *tim*^*sc*^ flies (Martin Anduaga et al., 2019) as well as during cold temperatures (<18°C) in natural conditions, suggesting that the TIM-COLD protein plays a role in seasonal adaptation (Montelli et al., 2015). Even lower temperatures (<14°C) in combination with short photoperiods induce diapause or reproductive dormancy in *D. melanogaster* (Saunders and Gilbert, 1990). It has recently been shown that accumulation of the cotranscription factor EYES ABSENT (EYA) is required for an efficient transition to female diapause (Abrieux et al., 2020). Interestingly, TIM-SC is the major Tim isoform under 10°C, and presumably binds to, and stabilizes EYA under these conditions, thereby promoting diapause entry.

Alternative *tim* splicing is directly influenced by the base composition of the respective *tim* introns and their interaction with specific splicing factors (Shakhmantsir et al., 2018; Foley et al., 2019; Martin Anduaga et al., 2019). Down-regulation of the pre mRNA processing factor 4 (PRPR4), part of the tri-SNRP spliceosome, results in accumulation of the *tim-tiny* transcript and increased free-running period length (Shakhmantsir et al., 2018). In contrast, down-regulation of the alternative splicing factor P-element somatic inhibitor (PSI) results in an increase of *tim-cold* and *tim-sc* transcripts, while *tim-tiny* levels are reduced (Foley et al., 2019). These changes of *tim* transcript balance are associated with a shortening of the free-running period, and an altered activity phase during temperature cycles. While PSI does not affect the temperature-sensitivity of the various *tim* splicing events, the circadian function of PSI depends on *tim* splicing. This is because flies in which a *tim* loss-of-function mutant was rescued with a *tim* gene lacking all thermosensitively spliced introns, did not show a period shortening or phase difference after knock down of PSI (Foley et al., 2019).

Collectively, the studies on temperature-dependent *per* and *tim* splicing point to a function in behavioral and physiological (reproductive dormancy) adaptation to different environmental conditions as experienced in temperate climates. There is only limited information about the potential role of alternative splicing in the daily entrainment to temperature cycles. While PSI is required to set the correct activity phase during temperature cycles, it is not required for synchronization to these cycles, suggesting a role in adaptation rather than entrainment. Nevertheless, it is possible that temperature-dependent splicing events of clock gene RNAs within the clock neurons directly contribute to temperature entrainment and it will be of interest to see if mutations that abolish the generation of the various temperature-dependent *per* and *tim* isoforms show defects in temperature entrainment.

## Clock Neurons in Isolated Fly Brains Are Largely Unresponsive to Temperature Changes

Based on the apparent minor contribution of cell autonomous temperature-dependent clock RNA alternative splicing events to temperature entrainment, it seems plausible that peripheral thermosensors, and/or temperature-sensitive neurons within the brain, signal temperature information to the clock neurons. To distinguish between these possibilities, cultured isolated brains have been exposed to LD and temperature cycles, to study if clock gene expression within the clock neurons can be synchronized to these cycles. LD exposure led to synchronized *period-luciferase* expression in isolated brains, presumably mediated by expression of CRY within subsets of the clock neurons (Sehadova et al., 2009; Roberts et al., 2015). In contrast, exposure of cultured tissues to temperature cycles was able to synchronize *period-luciferase* oscillations in peripheral clock cells, but not in central brain clock neurons (Sehadova et al., 2009). While these results do not exclude the existence of cell autonomous mechanisms operating in a small subset of clock neurons, or that some neurons receive temperature input from a temperature sensor located in the central brain, it seems clear that the majority of clock neurons depends on peripheral input for entrainment to temperature cycles. In support of this, it has been shown that subsets of the DN1p clock neurons can be activated by short cooling steps and inhibited by small heating steps, and that this requires the brain to be part of the intact fly (Yadlapalli et al., 2018). In cultured brains, the clock neurons did not respond to temperature changes, and in the intact fly, the neuronal responses depend on input from peripheral thermosensors located in both the body and the aristae (Yadlapalli et al., 2018).

## Sensory Structures Located in the Fly Body: A Role for the Chordotonal Organ in Temperature Responses

What are the peripheral sensory structures involved in circadian thermoreception and which molecules are involved in temperature sensing? A first clue to which structures might be involved came after a forward genetic screen that identified a mutant with defects in temperature entrainment (Glaser and Stanewsky, 2005). *nocte*^1^ (for *no circadian temperature entrainment*) was interesting because the mutant abolished *period-luciferase* oscillations during LL and temperature cycles (isolation phenotype), but oscillations were normal in LD and constant temperature (Glaser and Stanewsky, 2005). Similarly, behavioral synchronization to temperature cycles is severely impaired in LL, DD and LD, but *nocte*^1^ flies behave normally in LD and constant temperature (Glaser and Stanewsky, 2005; Sehadova et al., 2009; Chen et al., 2018). The *nocte* gene encodes a large (2300 amino acid) glutamine-rich protein of unknown function (Sehadova et al., 2009). *nocte*^1^ carries a point mutation that introduces a STOP codon after amino acid 1706 resulting in a truncated protein with severely attenuated function (Sehadova et al., 2009). *nocte* is widely expressed in the fly body and brain, with particularly strong expression in sensory organs of the PNS, including that of the Chordotonal organs (Cho). These are internal stretch receptors found between joints of limbs and within body segments (e.g.: within the femoral joints, base of the wings, halteres, and in the second segment of the auditory Johnston’s organ of the antennae) (Kim et al., 2003; Tuthill and Wilson, 2016). The mechanosensory neurons that make up the chordotonal organs are positioned within scolopidia – the fundamental units of the chordotonal organ. Each scolopidium contains accessory cells like dendritic cap cells and scolopale cells that protect and anchor 1–3 sensory neurons (Tuthill and Wilson, 2016). Most Cho mediate proprioception (Tuthill and Wilson, 2016; Mamiya et al., 2018) but some are also involved in sensing sound, gravity, and wind (Matsuo and Kamikouchi, 2013). More recently it has been shown that rhythmic or monotonous stimulation of Cho neurons, mediates circadian synchronization and vibration-induced sleep, respectively (Simoni et al., 2014; Öztürk-Çolak et al., 2020).

Interestingly, it was shown that RNAi mediated knockdown of *nocte* within the Cho mimicked the *nocte*^1^ behavioral phenotype in LL and temperature cycles (Sehadova et al., 2009). Moreover, both *nocte*^1^ and Cho-specific knockdown reduced the electrical responses of the more dorsally located DN1 neurons (DN1p) to acute temperature changes, and *nocte*^1^ interferes with synchronized PER and TIM expression in most clock neuronal subsets during temperature cycles (Chen et al., 2018; Yadlapalli et al., 2018), strongly implicating Cho as temperature sensors for clock neurons. In support of this, defects in Cho structure were present both in *nocte*^1^ and to a lesser extend in the hypmorphic *nocte*^*P*^ allele (Sehadova et al., 2009). Moreover, a temperature increase from 20 to 30°C induced movement-independent electrical responses in the leg nerve, which contains afferent projections from Cho neurons to the ventral nerve cord (VNC) (Chen et al., 2015). Finally, excitation of Cho neurons using rhythmic mechanical stimuli resulted in molecular and behavioral synchronization (Simoni et al., 2014). This synchronization was abolished in *nocte*^1^ and in mutations known to interrupt mechanosensing Cho function, strongly indicating a connection between Cho and clock neurons. Existing evidence strongly implicates Cho function in temperature sensing for circadian clock synchronization. While a direct proof of adult Cho neuron temperature-sensitivity is missing, larval Cho are temperature sensitive and double their firing frequency when heated from 15 to 30°C (Liu et al., 2003; Scholz et al., 2017). In addition, the postulated neuronal circuit connecting Cho neurons with clock neurons in the brain remains to be identified.

## Potential Thermoreceptors in the Chordotonal Organ

While it is very likely that Cho serve thermosensing functions in addition to their dominant role as proprioceptors, it is important to identify the molecular identity of the actual thermosensing molecules. Given the role of *nocte* for structural Cho integrity, it seems unlikely that this gene is important for thermosensing per se. Nevertheless, the NOCTE protein has been used to isolate interacting proteins, one of which belonged to the class of variant ionotropic glutamate receptors, IR25a (Chen et al., 2015). IR25a is expressed within subsets of the Cho neurons, and loss-of-function mutants fail to synchronize to temperature cycles both in LL and DD conditions. They show impaired synchronization of TIM oscillations within most subsets of the clock neurons, but particularly strong defects within the DN1 and DN2 (Chen et al., 2015), suggesting that theses neuronal groups receive temperature input from Cho neurons. Interestingly, entrainment defects of IR25a mutants are only observed in temperature cycles where the difference between night and day is <4°C, irrespective of the absolute temperature. This suggests that IR25a functions as an “amplitude-detector” rather than a thermometer, and that other thermosensors must exist to detect larger day/night temperature differences. That IR25a indeed functions as a temperature sensor in the Cho is supported by the fact that the movement-independent electrical responses to temperature increases in the leg nerve disappear in IR25a mutants. Moreover, ectopic expression of IR25a in the l-LNv clock neurons leads to temperature-dependent increase of neuronal firing, even if the temperature only increases by 2–3°C (Chen et al., 2015). While this is a strong indication that IR25a indeed acts as a thermoreceptor the temperature dependent increase in firing is quite moderate (Q_10_ > 4) compared to known thermoreceptors, as for example the thermo TRP channel TRPA1, whose ectopic expression in l-LNv clock neurons resulted in temperature-dependent increase of firing with a Q_10_ > 12 (Chen et al., 2015). It is possible that a sensor with weak temperature sensitivity is well suited as a circadian thermoreceptor. In contrast to a receptor that functions in fast avoidance responses to excessive heat or cold, temperature entrainment of the circadian clock requires responses to relatively small temperature changes over a long periods of time (hours) to allow reliable differentiation between night and day. In fact, fast responses to temperature changes, like those mediated by TRPA1, would presumably counteract circadian entrainment, because rapid temperature changes during the day, caused for example by sudden cloud cover or onset of rain, would activate such receptors.

**FIGURE 1:**
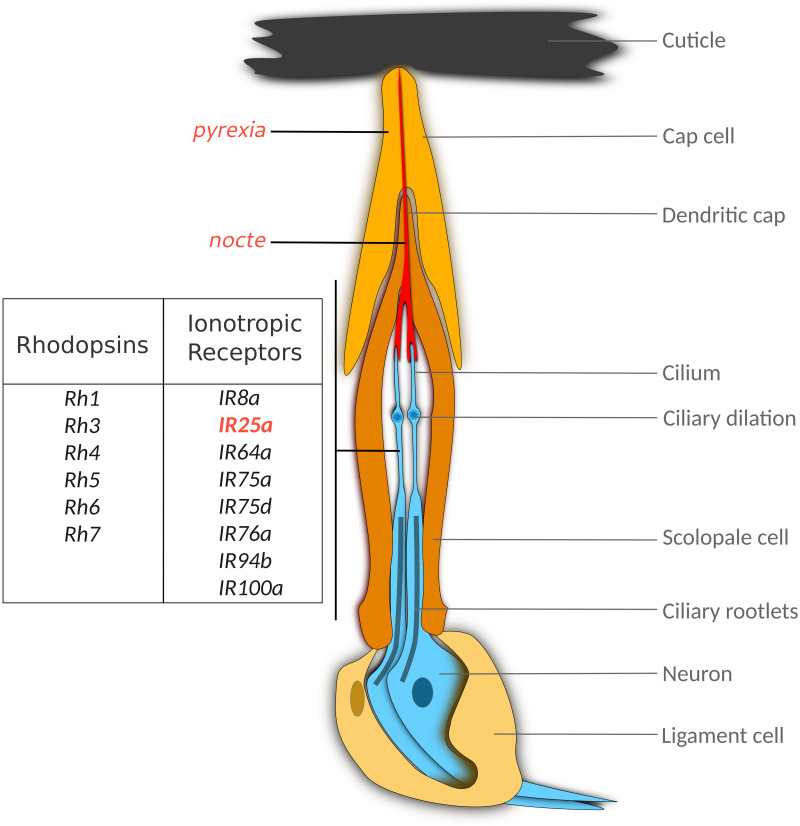
Thermoreceptor candidates in the scolopidium, the basic unit of Chordotonal organs. Larval and adult scolopidia consist of three major cell types, (i) the bipoloar Cho neuron(s), (ii) the scolopale cell, which encloses the dendrites and forms an endolymph-filled extracellular space, and (iii), the cap cell, which connects the neuron with the cuticle. While the main function of this unit is the conversion of mechanical vibration to electrical neuronal signals, the genes indicated to the left may contribute to thermal responses described for larval and adult Cho. While *nocte* mutations disrupt the structure of the dendritic cap, it is not clear whether the *pyx, Rh* and *IR* genes fulfill structural or sensory roles (or both). Genes that have been implicated in synchronization to temperature cycles are indicated in red. See text for details.

Another potential thermoreceptor expressed in Cho is the TRPA channel PYREXIA (PYX). In contrast to *IR25a* and *nocte*, *pyx* is not expressed in Cho neurons, but in so-called cap cells, which connect the dendritic cilia of the Cho neuron with the cuticle (Figure 1; Wolfgang et al., 2013). The same distribution of *pyx* expression is found in Johnston’s organ, the prominent Cho in the fly antennae, responsible for fly hearing and gravity sensing (Sun et al., 2009). Loss of *pyx* function results in impaired behavioral synchronization to 16°C:20°C temperature cycles both in LL and DD conditions (Wolfgang et al., 2013), along with altered PER synchronization in subsets of the clock neurons (including DN1 and s-LNv) (Roessingh et al., 2019). Interestingly, synchronization to warmer temperature cycles (e.g., 25°C:29°C) is normal (Wolfgang et al., 2013), indicating that PYX covers the low end of Drosophila’s physiological temperature range, and that other receptors are responsible for sensing warmer temperatures. It is surprising that PYX function is required at low temperatures, given that this TRPA channel functions to protect the fly from extreme heat (>40°C) and is activated at these temperatures when expressed heterologously (Lee et al., 2005). Moreover, *pyx* mutants lack morning and evening activity peaks under seminatural LD combined with 25°C:35°C temperature cycles, indicating that this channel operates over a large range of temperatures (Green et al., 2015). While it is possible that PYX expression in Cho cap cells contributes to temperature entrainment, recent findings suggest that neuronal *pyx* expression in the fly antennae plays an important role for clock synchronization (see below and Roessingh et al., 2019).

Other molecules potentially contributing to circadian temperature sensing within Cho include the TRPV channel INACTIVE (IAV). Structural integrity of larval Cho is required for choosing the preferred temperature (18°C) over cooler (14°C) temperatures (Kwon et al., 2010). Furthermore, the same study shows that expression of IAV within the larval Cho is required for this response, implicating this TRPV channel in temperature sensing or signaling. The same group showed that most of the Rhodopsin photopigments surprisingly also have temperature sensing capabilities within TRPA1 expressing cells in the larval body wall, the brain, and the VNC (Shen et al., 2011; Sokabe et al., 2016). For example, larvae mutant for the major fly Rhodopsin 1 were not able to choose the ideal temperature (18°C) compared to other comfortable temperatures (19–24°C), and this function could be replaced by substituting Rh1 with any other fly Rhodopsin (Rh2-Rh6), apart from Rh3 (Rh7 had not been identified then), and even by mouse circadian photopigment MELANOPSIN (Shen et al., 2011). Interestingly, two crucial components of the visual transduction cascade, the alpha subunit of the G_q_ protein and PLC-ß, encoded by the *norpA* gene, are also required for larval temperature discrimination, as well as TRPA1, which is presumably regulated by this cascade (Shen et al., 2011; Sokabe et al., 2016). Recently, it was found that Rh1 and Rh6 are also expressed within larval Cho neurons, where they fulfill proprioceptive functions by supporting the structural integrity of Cho neuron dendrites (Zanini et al., 2018). It seems therefore possible that Rhodopsins play a role in temperature synchronization, either as structural requirements for Cho integrity (similar to *nocte*), or as actual thermosensors in other cell types (see below).

## Thermoreceptors Located in the Fly Antennae

**FIGURE 2:**
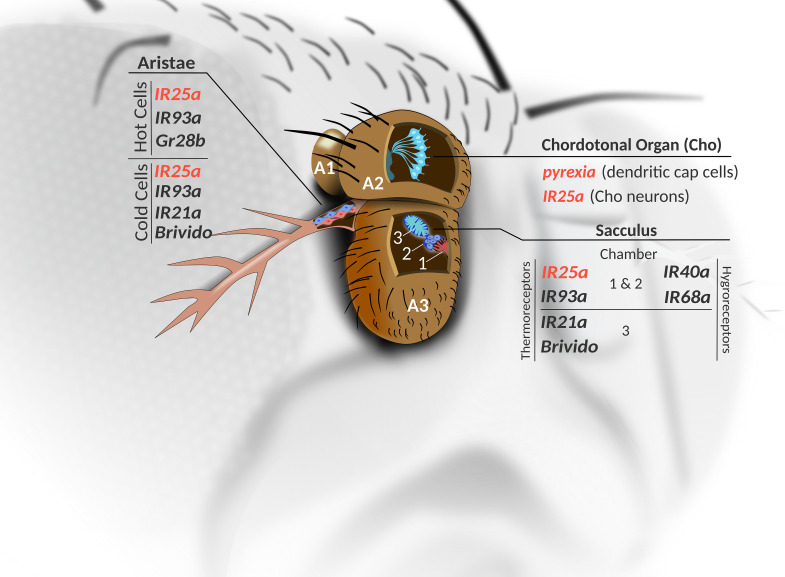
Thermoreceptor candidates of the antennae. Cross-sectional view shows the three segments of the antennae (A1–A3). Within the antennal Cho (JO) in A2, in addition to the thermo sensitive IR25a, seven other IRs are expressed in subsets of the Cho neurons (see Figure 1), while *pyx* expression is found within the non-neuronal dendritic cap cells (see also Figure 1), as well as in a group of unidentified neurons within antennal segments 1 and 2 (not shown). Antennal IRs involved in sensing temperature and humidity innervate different chambers of the sacculus (located in A3). In addition, three hot (red) and three cold (blue) cell bodies are located at the base of the aristae in A3. Genes expressed in the hot and cold cells are indicated. IR: Ionotropic Receptor. Gr28b: gustatory receptor 28b. Brv: Brivido. Red font indicates genes with a known function in temperature entrainment.

The antennae is an important thermosensory organ, housing several sensory structures that help the fly detect temperature changes and confer protection against adverse environmental conditions. In the second antennal segment, cells of the largest stretch-activated Cho of the fly, known as the Johnston’s organ (JO) express Rh3, 4, 5 and 6, with Rh5 and Rh6 being important for hearing (Senthilan et al., 2012). Similarly, seven IR genes are expressed in JO (IR8a, 64a, 75a, 75d, 76a, 94b, 100a), and only IR75a has been linked to hearing, while IR64a and IR94b are not required for normal responses to acoustic stimuli (Figure 1) (Senthilan et al., 2012). While a role for the Rhodopsins in temperature preference has only been shown in larvae (Shen et al., 2011; Sokabe et al., 2016), it seems possible that a subset of the Cho-expressed Rhodopsin and IR receptors participate in thermal clock resetting (Figures 1, 2). Moreover, the non-neuronal dendritic cap cells of the Cho express *pyrexia* (*pyx*) (Figure 1), which may also contribute to temperature entrainment (Sun et al., 2009; Wolfgang et al., 2013; Roessingh et al., 2019).

Thermoreceptors are also found in the sacculus – a multi-chambered invagination in the third segment of the fly antennae (Shanbhag et al., 1995). Ionotropic receptor (IR) expression is found in neurons innervating all three chambers of the sacculus. These receptors belong to the variant ionotropic glutamate receptor family (iGluR) and have been more extensively described for their role in chemo-sensation. Importantly, the neurons expressing the temperature sensitive IR25a and IR93a innervate chambers 1 and 2, while IR21a expression is found near chamber 3 (Benton et al., 2009; Abuin et al., 2011). On the other hand, the IRs functioning in hygrosensation (IR40a and IR68a), surround chambers 1 and 2 (Figure 2) (Enjin et al., 2016; Frank et al., 2017). Interestingly, IR25a and IR93a are co-expressed with IR40a here, and are also required for humidity responses (Enjin et al., 2016). The cooling-sensors Brivido 1-3 also function within the cold cells of sacculus chamber 3 (Gallio et al., 2011), as well as in the three cold cells at the base of the aristae – a feather-like extension from the third antennal segment. Flies with impaired function in any of the three *brivido* genes lose their rapid and extremely sensitive responses to cooling steps (Gallio et al., 2011). However, a more recent study indicates that these cold sensors are not required for temperature avoidance behaviors (Budelli et al., 2019).

Instead, expression of three different IRs in the three hot and three cold cells found at the base of the arista is required for mediating thermotactic behavior (Budelli et al., 2019). They first establish that the hot and cold cells do not function as hot and cold sensors, respectively, but can each respond to temperature changes in both the cool and warm range. Electrophysiological recordings show that hot cell responses are slow-adapting in nature, with firing levels correlating with the temperature increase (Q10∼4.4). Conversely, hot cells are inhibited by cooling. On the other hand, cold cells display transient activation in response to cooling and are inhibited by warming. IR25a and IR93a are found in both hot and cold cells, while IR21a expression is confined to the three cold cells. The hot cells also express the gustatory receptor Gr28b (Thorne and Amrein, 2008; Ni et al., 2013), which is necessary for warm responses when the rate of temperature increase is slow (∼1°C per second) (Liu et al., 2015). This warm receptor also plays a role in mediating avoidance of warm temperatures, albeit with less influence than warm avoidance mediated by IR21a expressed in the cold cells (Budelli et al., 2019).

Using transmission electron microscopy for a closer examination, it was shown that the characteristic layers of membrane lamellae of cold cell outer segments, and the “bossy” orthogonal surface substructures (BOSSs) between each layer were absent in IR93a, IR25a and IR21a mutants, with the most dramatic defects seen in the absence of the cold-cell exclusive IR21a (Budelli et al., 2019). These morphological defects likely compromise the efficiency of thermoreception in these cells, and this might explain why the mutants lose their ability to mediate thermotactic behavior. Additionally, ectopically expressing IR21a in hot cells not only allowed for cold-sensing activation in these cells (Ni et al., 2016; Budelli et al., 2019), but it also altered hot cell sensory endings so that they became enveloped by the cold cell’s sensory endings, with BOSS structures between each outer segment membrane (Ni et al., 2016; Budelli et al., 2019). Considering these findings, it raises the question as to how exactly these IRs mediate temperature sensation. Are the receptors themselves thermoreceptive, or do they play a more indirect role where they are merely needed for contributing to the morphogenesis of the dendritic endings of sensory neurons? Ectopic expression of IR25a in the l-LNv conferred thermosensitivity to these clock neurons, which are normally imperceptive to temperature input, indicating that at least this IR may directly sense temperature (Chen et al., 2015). Irrespective of whether the IRs in the aristae are directly thermosensitive, it is clear that without the thermosensitive cells in the arista, the normal responses of the dorsal DN1p clock neurons are blunted, and the timing of sleep in Drosophila is altered during temperature cycles (Yadlapalli et al., 2018). Neither the thermosensitive molecules, nor the pathway that is involved to send input to the DN1p have been described.

Confirming if these temperature-sensitive receptors also play a role in entraining circadian rhythms to recurring temperature changes is still an ongoing and challenging effort. Of the potential temperature-sensing molecules in the antennae, only PYX has been shown to contribute to temperature entrainment. As discussed above, *pyx* mutants cannot entrain to temperature cycles in the cooler range (16°C:20°C) (Wolfgang et al., 2013). In addition to non-neuronal cap cell expression described above, *pyx* is also expressed within unidentified neurons of the 2nd and 3rd antennal segment (Sun et al., 2009; Tang et al., 2013; Roessingh and Stanewsky, 2017). Using a transcriptional reporter of intracellular calcium (TRIC) coupled to luciferase for bioluminescence-based reporting, *pyx* mutant cells exhibited higher intracellular calcium levels specifically during temperature cycles, indicating abnormally high neuronal excitation levels (Roessingh et al., 2019). The authors hypothesized that mutant PYX channels result in defective signaling, which consequently affects synchronization of the PDF^+^ s-LNVs and DN1 clock neuron subgroups. Consistent with this idea, removing these malfunctioning PYX channels by ablating the antennae, improved behavioral synchronization to temperature cycles (Roessingh et al., 2019). The circuit mediating temperature entrainment using input from the pyrexia expressing cells in the antennae has not been described.

**FIGURE 3:**
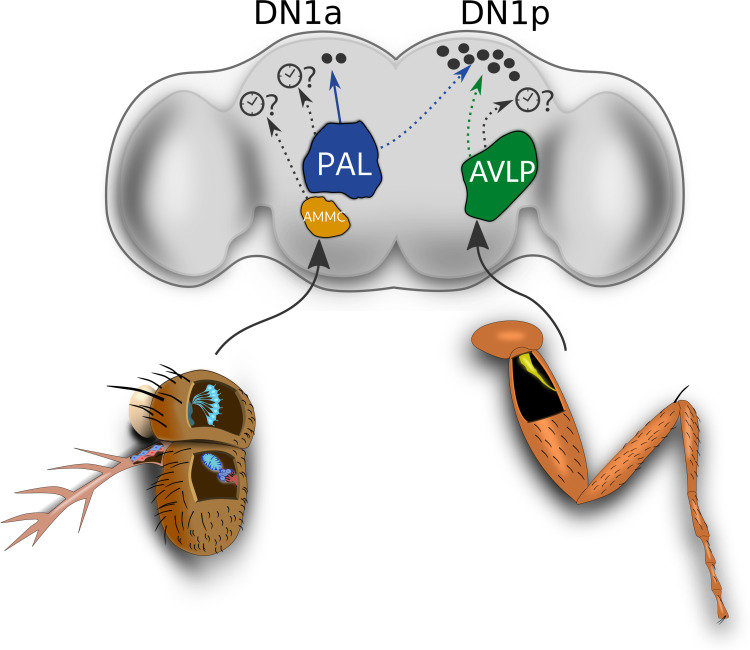
Circuits potentially involved in circadian clock resetting by temperature. Temperature input from the thermoreceptive neurons of the sacculus and the aristae terminate in the PAL (shown in blue). From the PAL, a circuit for responses to absolute cold temperatures (below 25°C) to the DN1a clock neurons has been mapped (thermosensory projection neuron shown in the solid blue line). Other predicted thermo circuits from the PAL to the clock neurons are represented as black dotted lines. The antennal ChO neurons are known to target the AMMC (shown in orange), while the leg Cho neurons ascending from the leg neuropil of the VNC (not shown), target the most ventral region of the AVLP (shown in green) (Tsubouchi et al., 2017). Input from the *nocte* expressing Cho neurons in the body (e.g., the leg Cho), as well as from the thermoreceptive cells in the aristae mediate DN1p responses to brief temperature changes via unknown circuits (blue and green dotted lines). Other potential thermosensory circuits arising from the AMMC and the AVLP that could target other central clock neurons are represented as black dotted lines. This could, for example, include a circuit encoding input from the IR25a-expressing neurons within the leg Cho neurons, which mediate behavioral synchronization to shallow temperature cycles. DN1p: Posterior Dorsal Neuron 1. DN1a: Anterior Dorsal Neuron 1. PAL: Posterior Antennal Lobe. AVLP: anterior ventrolateral protocerebrum. AMMC: antennal mechanosensory and motor center.

The hot and cold cells of the aristae are known to target hot and cold glomeruli situated in the posterior antennal lobe (PAL), respectively (Frank et al., 2015) (Figure 3). The cold glomerulus also receives input from the cold cells in the sacculus. On the other hand, the *pyx* expressing neurons of the 2nd antennal segment are suggested to send input to the TRPA1 expressing neurons (AC neurons), and this input ultimately reaches the hot glomerulus of the PAL (Gallio et al., 2011; Tang et al., 2013). Later, it was shown that this region also receives dry and humidity signals sensed by hygrosensitive cells in the antennae (Frank et al., 2015), so that the sensory map of the PAL resembles a “hub” from which dendrites of ascending projection neurons (PNs) can extract and integrate information about temperature and humidity for sending to higher brain centers.

Three primary tracts ascending from PAL PNs take different paths but ultimately target the same higher brain centers like the lateral horn, the calyx of the mushroom body and the posterior lateral protocerebrum (PLP), all of which are important sensory processing centers (Heisenberg, 2003; Frank et al., 2015). It remains a question of interest to identify if any of these pathways carry information about daily temperature cycles from the antennae to entrain the clock neurons.

More recently, the complete circuit of one of the previously described PNs (R60H12, also referred to as thermosensory projection neuron TPN-II), starting from the peripheral temperature sensitive cells of the antennae, all the way to the two more anteriorly positioned DN1 clock neurons, DN1a, has been revealed (Alpert et al., 2020). Live calcium imaging and electrophysiological recordings confirmed that TPN-II is oblivious to heating steps, and only respond in a slow-adapting manner to absolute cold temperatures (below 25°C) with firing rates correlating with the absolute temperature reached. Notably, TPN-II innervates the PAL’s cold glomerulus and collects input from three distinct cell types mediating detection of absolute cold temperatures. These include the chamber one cells of the sacculus, the cold cells in the aristae and interestingly – a new IR25a-expressing neuron within the head capsule. This latter thermosensitive neuron bears resemblance to the TRPA-1 anterior cell (AC) neurons, which also sit at the edge of the antennal nerve at the most anterior surface of the brain (Hamada et al., 2008). This finding suggests the existence of other anteriorly positioned neurons (i.e., novel AC neurons) expressing diverse thermoreceptive molecules. Of the three cell types that were shown to elicit slow-adaptive responses to absolute cold temperatures and to cooling steps, only the sacculus neurons exclusively responded to absolute cold temperatures, suggesting that they may be the primary contributors for TPN-II activity.

Importantly, this work showed that input from the cold thermosensors in the antennae was needed for inhibition of DN1a activity to allow normal temporal adaptation of sleep-wake patterns in a cold environment (Alpert et al., 2020). Taking this into consideration, TPN-II holds potential to also be involved in mediating temperature entrainment, since its slow-adapting manner of response to temperature input makes it ideal for resetting behavioral patterns in response to recurring temperature cycles. This work raises many other questions: Would separate TPNs responding to absolute warm be needed for temperature entrainment? Would warm-and cold-excited TPNs target the same clock neurons and if not, how would information about warm and cold signals be integrated by different oscillators to produce synchronization of locomotor activity? What is clear at this point is that much remains to be uncovered about the circuitries that contribute to temperature entrainment.

Despite the potential role for the antennal thermosensors in temperature entrainment, it has been clearly shown that flies can still entrain to temperature cycles without their antennae (Sehadova et al., 2009; Chen et al., 2015; Roessingh et al., 2019), meaning that the antennae and all its thermoreceptors are not required for temperature entrainment. Nevertheless, there are pathways carrying temperature input from the antennae that may actively contribute to temperature entrainment in the fly (Yadlapalli et al., 2018; Roessingh et al., 2019). Identifying the circadian thermoreceptors and mapping out these antennal circuits are still an important part of understanding temperature entrainment in *Drosophila*. Additionally, the dispensability of the antennae supports the existence of alternative thermosensory circuits, including Cho located in the body, and perhaps other thermosensors in the PNS or internal thermoreceptors within the head capsule (e.g., novel AC neurons) that are contributing to temperature entrainment in the absence of the antennae. Finally, one could consider drawing similarities to light entrainment. Both, the photoreceptors in the compound eyes and Cryptochrome in a portion of clock neurons, are known to mediate normal synchronization of the brain clock to light–dark cycles. No obvious light entrainment defects are observed if one of the two systems is impaired (see below and Stanewsky et al., 1998), and this supports the notion that the antenna is still a candidate thermosensory organ for mediating temperature entrainment.

## TRPA1

The involvement of TRPA1 in synchronization to temperature cycles is controversial. While it is clear that TRPA1 mediates an astonishing array of temperature-dependent behaviors during larval development and in adults (e.g., sleep, temperature preference, see above, and (Roessingh and Stanewsky, 2017) for a comprehensive review of TRPA1’s role in temperature dependent behaviors), a prominent role in daily temperature entrainment can be excluded. Several TRPA1 isoforms with different thermal properties exist (Bellemer, 2015). Temperature activation of TRPA1 can occur either directly (between 24 and 36°C depending on the isoform) or indirectly, at temperatures below 24°C, downstream of G_q_ and PLC-ß signaling (see above and Bellemer, 2015). This complexity is likely responsible for the multitude of TRPA1 functions in regulating temperature-dependent behaviors and at the same time makes it difficult to assign functions to specific TRPA1 isoforms and their underlying activation mechanisms. With regard to temperature entrainment, it seemed possible that TRPA1 expression within thermosensitve “AC” neurons (Hamada et al., 2008) would contribute to temperature entrainment. But although they contact the s-LNv clock neurons, silencing of AC neurons or depleting them of TRPA1 did not prevent synchronization to DD 20°C:25°C or 25°C:29°C temperature cycles (Tang et al., 2017). Different *trpA1* reporter lines reveal varying degrees of *trpA1* expression in subsets of the clock neurons, although antibody staining or other in situ approaches have not confirmed this (Hamada et al., 2008; Lee and Montell, 2013; Yoshii et al., 2015). Nevertheless, analysis of *trpA1* mutants during 18°C:29°C temperature cycles in DD revealed only very subtle effects on behavioral temperature entrainment and PER oscillations within clock neurons (Lee and Montell, 2013). Subsequently, two independent studies concluded that TRPA1 is not required for temperature entrainment in 16°C:25°C, 20°C:29°C, and 21°C:29°C temperature cycles in DD (Roessingh et al., 2015; Das et al., 2016). Interestingly, the same studies revealed a role for TRPA1 in repressing day activity (the “siesta”) during 20°C:29°C temperature cycles, which may have contributed to the initial interpretation that TRPA1 mediates temperature entrainment (Lee and Montell, 2013). In summary, while it is well established that TRPA1 regulates multiple temperature dependent behaviors during larval stages and in the adult fly (Roessingh and Stanewsky, 2017), temperature entrainment of the circadian clock appears to be one prominent exception.

## Clock Neuronal Targets of Peripheral Temperature Entrainment

Several lines of evidence point to the DN1 group of clock neurons as one important “hub” for receiving temperature input from the periphery. First, DN1p are connected to two distinct sets of thermosensory neurons expressing TRPA1, which regulate the later onset of the siesta at very high temperatures (≥29°C) (Lamaze et al., 2017). Second, the DN1a are connected to a cold sensitive thermosensory circuit originating in the fly aristae (Alpert et al., 2020). The DN1a are inhibited by cold temperature (18°C) and likely contribute to the adaption of behavior to cold conditions (Zhang et al., 2010; Alpert et al., 2020). Interestingly, the sLNvs receive input from the DN1a neurons via CCHamide signaling for proper timing of sleep and activity, indicating that DN1a mediated temperature input could influence other clock neurons (Fujiwara et al., 2018). Third, subsets of the DN1p neurons are activated by brief cold, and inhibited by brief warm temperature changes, most likely related to their function of timing sleep during natural temperature cycles (Chen et al., 2018; Yadlapalli et al., 2018). As discussed above, DN1p temperature responses depend on the presence of intact peripheral thermosensors present in the aristae and Cho organs located in the body (Figure 3) (Yadlapalli et al., 2018). It seems therefore logical to assume that peripheral sensory input to the DN1 neurons also mediates synchronization to temperature cycles. Indeed, synchronization of TIM or PER expression in the DN1 was significantly altered in *IR25a*^–^, *nocte*^1^, and *pyx*^3^ mutants (Chen et al., 2015, 2018; Roessingh et al., 2019), strongly supporting a role for this neuronal group in receiving temperature input from multiple peripheral thermosensors. Additionally, impairing DN1p neuronal activity affected temperature entrainment to both rectangular and gradually ramped temperature cycles (Chen et al., 2015; Yadlapalli et al., 2018). Further support for this came from a recent study analyzing the function of the daily plasticity changes of the dorsal s-LNv projections (extend and spread of arborizations), which are observed under constant temperature (LD and DD, Fernández et al., 2008) and, although less pronounced, also under temperature cycles (Fernandez et al., 2020). These projections harbor both pre-and postsynaptic structures, suggesting that apart from the prominent PDF-release dependent output functions, s-LNv dendritic projections in the dorsal brain may also receive signals from other neurons (Yasuyama and Meinertzhagen, 2010). During maximum arborization in the early morning, dorsal PDF-containing s-LNv projections are tightly intermingled with DN1p projections (Fernandez et al., 2020), and direct contacts between both cell types have been reported (Guo et al., 2016). Interestingly, abolishing the daily dorsal spreading of the s-LNv arborizations (and thereby presumably a direct connection between DN1p and s-LNv), resulted in an altered locomotor activity pattern during ramped 20°C:28°C temperature cycles, while behavior under constant temperature (LD and DD) was normal (Fernandez et al., 2020). Subsets of the DN1p release glutamate (Hamasaka et al., 2007; Guo et al., 2016), and mutations in both ionotropic and metabotropic glutamate receptors phenocopied the behavioral pattern of flies with depleted s-LNv arborizations (Fernandez et al., 2020). It therefore seems likely that during temperature cycles, the DN1p inhibit s-LNv activity via glutamate signaling in the early morning. While flies with impaired s-LNv plasticity clearly show altered behavioral patterns during temperature cycles, PER oscillations within their s-LNv were synchronized, albeit with reduced amplitude. Moreover, the behavioral rhythms were still entrained to the temperature cycle, and mainly showed a reduction of the main activity peak normally observed at the end of the warm phase (Fernandez et al., 2020). Therefore, it seems clear that other mechanisms must exist for synchronizing the s-LNv to temperature cycles. For example, it has been shown that the sLNv receive synaptic input from AC neurons, which is important for the regulation of the daily temperature preference rhythm (TPR), although AC neuron silencing did not impair temperature entrainment and various conditions (see above and Tang et al., 2017). Likewise, other clock neurons may receive input from other thermosensors and the DN1 could also regulate activity of non s-LNv clock neurons, like the LNd (Guo et al., 2016).

## Other Factors Involved in Temperature Entrainment

To date there are only a few other genes that have been implicated in temperature entrainment of the fly clock. Flies lacking the phospholipase C-ß, (PLC-ß) encoded by the *norpA* gene, were found to block synchronization of *per-luc* expression and behavioral rhythms to temperature cycles in LL (Glaser and Stanewsky, 2005). This gene is also responsible for blocking alternative splicing of *dmpi8* in cold conditions, resulting in a “cold-locked” splicing mode in *norpA* mutants even during warm temperatures and long photoperiods (Collins et al., 2004; Majercak et al., 2004). When tested during temperature cycles in LL, levels of spliced *per* mRNA were higher in the *norpA* mutant flies compared to controls as expected (Glaser and Stanewsky, 2005, 2007). Interestingly, while always higher than in wild type controls, the ratio of spliced to unspliced *per* RNA in *norpA* mutants was not constant during the temperature cycle, but peaked at the end of the cold and beginning of the warm phase (morning), with a clear trough at the transition from warm to cold (evening) (Glaser and Stanewsky, 2005, 2007). Nevertheless, because there were no significant *per* splicing ratio oscillations observed in wild type control flies, it remains questionable if *per* splicing contributes to daily resetting to temperature cycles. While it seems well established that *norpA-*mediated regulation of *per* splicing contributes to seasonal adaptation (Collins et al., 2004; Majercak et al., 2004; Breda et al., 2020), the role of *norpA* in daily temperature entrainment remains elusive, potentially restricted to LL (Chen et al., 2020).

Phosphorylation of the transcription factor CLOCK (CLK) was also found to be important for daily temperature entrainment. Interestingly a mutant *Clock* gene, in which 15 potential serine phosphorylation sites are mutated to alanine, is able to support circadian rhythmicity in LD cycles and DD, while synchronization to temperature cycles is impaired. Both, rhythmicity in constant conditions, after exposure to temperature cycles as well as behavior during such cycles are affected, suggesting that rhythmic temperature signals ultimately influence CLK phosphorylation and thereby the levels and activity of this key circadian transcription factor (Lee et al., 2014).

Finally, in the mouse liver, heat shock factor 1 (HSF1) regulates rhythmic gene expression, including that of *mper2*, during temperature cycles, providing a potential mechanism for temperature entrainment of peripheral clocks in mammals (Saini et al., 2012). Based on these findings, and the observation that components of the HSP/HSP90 are rhythmically expressed in fly heads during temperature cycles (Boothroyd et al., 2007) the potential involvement of *Hsp90* in temperature entrainment was studied (Goda et al., 2014). While there was no clear effect of *Hsp90* mutants on molecular entrainment of *per* and *tim* gene products in whole fly heads, loss of *Hsp90* function surprisingly resulted in faster behavioral resetting to advanced (but not to delayed) temperature cycles (16°C:28°C). While this could point toward a specific role for *Hsp90* in clock neurons, the altered behavioral patterns during temperature resetting more likely reflect the increased behavioral plasticity of *Hsp90* mutants (Hung et al., 2009).

## Synchronization of Peripheral Clocks

There are numerous reports demonstrating that peripheral clocks of the fly can be synchronized to temperature cycles. Most studies used protein or RNA extracts from fly heads, which mainly report clock gene expression in the compound eye photoreceptors, which are considered to contain peripheral clocks. These studies show that clock gene expression can be synchronized to temperature cycles both in LL and DD, both at the RNA and protein level (Stanewsky et al., 1998; Glaser and Stanewsky, 2005; Yoshii et al., 2005; Boothroyd et al., 2007; Martin Anduaga et al., 2019). In addition, whole-fly bioluminescence expression emanating from *period* and *timeless-luciferase* transgenes, which report clock genes expression in abdominal and eye clocks, are robustly synchronized by temperature cycles (Glaser and Stanewsky, 2005; Sehadova et al., 2009; Harper et al., 2017). Interestingly, while CRY is important for light entrainment of peripheral clocks (Ivanchenko et al., 2001), CRY is not required for temperature synchronization of clock gene cycling in the periphery – at least when measured in the context of the intact fly (Stanewsky et al., 1998; Glaser and Stanewsky, 2005; Harper et al., 2017). With regard to actual biological rhythms regulated by peripheral clocks, CRY’s role appears ambiguous: While the adult cuticle deposition rhythm can be synchronized to temperature cycles in the absence of CRY (Ito et al., 2011), electroantennogram (EAG) rhythms in the fly antennae, which reflect the daily changes of olfactory sensitivity, cannot (Krishnan et al., 2001). Most likely, this discrepancy is related to an additional, light-independent function of CRY as central clock factor in subsets of the peripheral clocks (Collins et al., 2006).

The experiments described above do not reveal if peripheral clocks can be entrained without a functional connection to the central clock in the fly brain. To address the autonomy of peripheral clock entrainment by temperature cycles, isolated fly tissues (legs, wings, heads, abdomen, proboscis, halteres, and antennae) of *per-luc* and *tim-luc* flies have been exposed to temperature cycles in culture (Krishnan et al., 2001; Glaser and Stanewsky, 2005; Sehadova et al., 2009). These experiments clearly showed that peripheral clocks of all tissues can be synchronized in an at least a tissue autonomous way. While CRY is not required for peripheral clock entrainment by temperature in whole flies (see above), this has not been conclusively addressed in isolated peripheral clocks: Tissues of *per-luc* and *tim-luc cry^*b*^* flies were entrained to temperature cycles, and subsequently measured in constant temperature in DD (Krishnan et al., 2001; Levine et al., 2002). Compared to *cry*^+^, the number of rhythmic *cry*^*b*^ tissue samples was significantly reduced, which mostly likely simply reflects the requirement of CRY for maintaining circadian clock function in peripheral clocks. It remains to be determined if CRY is required for peripheral clock oscillations in isolated tissues during temperature cycles.

It seems likely that cell-autonomous mechanisms mediate peripheral clock entrainment to temperature cycles, perhaps involving some of the temperature-dependent alternative splicing events discussed at the beginning of this article. Nevertheless, all tissues tested so far are located at the surface of the fly; therefore, contribution of sensory thermoreceptors cannot be excluded. It is noteworthy however, that constant light-induced arrhythmic sperm release and transfer in the abdomen of the moth *Spodoptera littoralis* is restored by temperature cycles (Syrova et al., 2003).

## Difficulties Studying Temperature Entrainment

From our discussion above, several problems associated with the study of temperature entrainment are obvious. Over a wide range of physiological temperatures, (16–29°C, outside the range of temperatures inducing diapause or heat stress) temperature cycles with a small amplitude (2°C difference between day and night) serve as a potent Zeitgeber for entrainment. It appears that different thermo receptors operate in different temperature intervals (e.g., PYX in the lower range), and that some receptors are able to detect temperature differences over the entire spectrum (e.g., IR25a). It follows, that in order to determine the contribution of a candidate receptor, a multitude of entrainment conditions needs to be tested (e.g., PYX would not have been found if only 20–29°C temperature cycles had been used). Moreover, there is the possibility and likelihood of redundancy as observed in light entrainment. For example, it is difficult to observe entrainment defects of *cry* mutants in standard LD cycles, due to the contribution of the compound eyes (Stanewsky et al., 1998). Only by applying more complex entrainment regimes (e.g., shifts of the LD cycles, light intensity changes) or by combining different mutants (e.g., visual system and *cry*), the contribution of each system is revealed. Therefore, care must be taken before ruling out the contribution of a certain factor or molecular mechanism (for example the alternative splicing mechanisms discussed at the beginning of this article) to temperature entrainment. In addition, a powerful assay for studying light entrainment is not really working for temperature: depending on the time of day, brief light-pulses elicit robust behavioral and molecular phase advances or delays, giving rise to informative phase response curves (PRCs). In contrast, comparable temperature pulses are basically incapable of inducing phase shifts, at least as long as they are within the physiological range (e.g., Busza et al., 2007). While this makes sense from a biological perspective (it would be disadvantageous if sudden weather-inflicted temperature changes would serve as stable resetting cues), it mitigates against a rapid and efficient analysis of potential new temperature entrainment factors.

Finally, constant light (LL) served as a powerful tool for identifying factors acting in the light input pathway (e.g., Emery et al., 2000; Peschel et al., 2006; Dubruille et al., 2009). When it comes to testing if a genetic variation is required for synchronizing the clock to temperature cycles, it makes sense to eliminate the other main *Zeitgeber* (LD cycles) – by keeping the light conditions constant. In DD temperature cycles synchronize behavioral activity peaks to occur in the first half of the thermophase (Gentile et al., 2013). Interestingly, while temperature cycles also robustly synchronize the circadian clock in LL, overriding the effects that normally lead to the constitutive degradation of TIM, (Glaser and Stanewsky, 2005; Yoshii et al., 2005) the behavioral activity peaks occur toward the second half of the thermophase (Gentile et al., 2013). While this (and temperature synchronization in LL) is an interesting and unresolved phenomenon as such, it adds another dimension to the problem of temperature entrainment and even more environmental conditions that need to be scrutinized when genetic variants are tested for their potential contribution. In other words, it is possible that mutants interfere with temperature entrainment in DD but not in LL and vice versa, which is why it is best to test the contribution of candidate genetic variants to temperature entrainment in both DD and LL conditions. While it could be argued that studying temperature entrainment in LL is a somewhat artificial construct, it may actually be a relevant condition experienced by animals (including some fly species) north of the polar circle in summer. While still being exposed to changes in light intensity and quality during the polar summer, temperature cycles may gain importance as more reliable Zeitgeber compared to light (cf., Harper et al., 2016).

## Conclusion and Outlook

It appears that the entrainment mechanisms have evolved to allow for fast behavioral responses to rapidly changing temperature changes to avoid immediately harmful conditions, while still using the relatively small temperature changes between day and night as potent Zeitgeber. Interestingly, and comparable to light-entrainment and direct effects of light on behavior, subsets of the clock neurons are used to integrate both modalities and to coordinate the appropriate behavior. While for temperature this involves the DN1p (see above and Lamaze and Stanewsky, 2019), the l-LNv have been shown to mediate both direct effects of light (e.g., arousal) and entrainment (Rosato and Kyriacou, 2008; Schlichting, 2020). Interestingly, both light and temperature entrainment employ cell-autonomous mechanisms and peripheral sensory organs for proper synchronization. In the case of light, this is accomplished by the compound eyes and CRY expressed in subsets of the clock neurons, as well as by CRY for synchronization of peripheral clocks. For temperature, it appears that the clock neurons mainly rely on peripheral sensory input, while peripheral clocks presumably employ an as yet unidentified cell-autonomous mechanism. From what is known so far, peripheral input to the central clock appears complex, involving various sensory structures and different families of thermos-receptive molecules (IRs and TRP channels). Future work will certainly extend the list of existing players and will shed light on the neuronal circuits connecting the peripheral sensors with the clock circuit. It will be particularly challenging to disentangle how the clock can distinguish sudden and potentially harmful temperature changes from the regular daily changes of average temperature it needs for clock resetting.

## Author Contributions

RG and RS wrote and edited the manuscript. Both authors contributed to the article and approved the submitted version.

## Conflict of Interest

The authors declare that the research was conducted in the absence of any commercial or financial relationships that could be construed as a potential conflict of interest.
